# Sputum microbiota as a potential diagnostic marker for multidrug-resistant tuberculosis

**DOI:** 10.7150/ijms.53492

**Published:** 2021-03-03

**Authors:** Dongzi Lin, Xuezhi Wang, Yanyun Li, Wei Wang, Yumei Li, Xiaolin Yu, Bingyao Lin, Yinwen Chen, Chunyan Lei, Xueying Zhang, Xilin Zhang, Juan Huang, Bihua Lin, Weiqing Yang, Jie Zhou, Jincheng Zeng, Xinguang Liu

**Affiliations:** 1 Dongguan Key Laboratory of Medical Bioactive Molecular Developmental and Translational Research, Guangdong Provincial Key Laboratory of Medical Molecular Diagnostics, Guangdong Medical University, Dongguan, China; 2 Department of Laboratory Medicine, Foshan Fourth People's Hospital, Foshan, Guangdong, 528041, China; 3 Department of Laboratory Medicine, Dongguan Sixth People's Hospital, Dongguan, Guangdong, 523008, China

**Keywords:** Tuberculosis, drug resistance, sputum microbiota, 16S rRNA sequencing

## Abstract

The prevalence of drug-resistant *Mycobacterium tuberculosis* (Mtb) strains makes disease control more complicated, which is the main cause of death in tuberculosis (TB) patients. Early detection and timely standard treatment are the key to current prevention and control of drug-resistant TB. In recent years, despite the continuous advancement in drug-resistant TB diagnostic technology, the needs for clinical rapid and accurate diagnosis are still not fully met. With the development of sequencing technology, the research of human microecology has been intensified. This study aims to use 16 rRNA sequencing technology to detect and analyze upper respiratory flora of TB patients with anti-TB drug sensitivity (DS, n = 55), monoresistance isoniazide (MR-INH, n = 33), monoresistance rifampin (MR-RFP, n = 12), multidrug resistance (MDR, n = 26) and polyresistance (PR, n = 39) in southern China. Potential microbial diagnostic markers for different types of TB drug resistance are searched by screening differential flora, which provides certain guiding significance for drug resistance diagnosis and clinical drug use of TB. The results showed that the pulmonary microenvironment of TB patients was more susceptible to infection by external pathogens, and the infection of different drug-resistant Mtb leads to changes in different flora. Importantly, seven novel microorganisms (Leptotrichia, Granulicatella, Campylobacter, Delfitia, Kingella, Chlamydophila, Bordetella) were identified by 16S rRNA sequencing as diagnostic markers for different drug resistance types of TB. Leptotrichia, Granulicatella, Campylobacter were potential diagnostic marker for TB patients with INH single-resistance. Delftia was a potential diagnostic marker for TB patients with RFP single drug-resistance. Kingella and Chlamydophila can be used as diagnostic markers for TB patients with PR. Bordetella can be used as a potential diagnostic marker for identification of TB patients with MDR.

## Introduction

With the development of sequencing technology and in-depth study on human micro-ecology in recent years, it has been found that microorganisms colonized in human body play an important role in human physiological processes^1^, and TB may involve complex microbial community interactions, rather than a result of single pathogen in conventional idea^2^-^6^. The broken dynamic balance of microecological composition and abundance in healthy individuals will lead to bacterial flora disorder, proliferation of pathogenic bacteria, organisms-associated pathological immune responses and disease generation^7^. Several studies have shown that opportunistic pathogens identified in sputum samples appear before the patient's treatment, indicating that the disorder of sputum flora is not a result of antibiotics^4^,^5^. It suggests that pulmonary micro-ecological environment of TB patients may be more susceptible to colonization by foreign microorganisms, and the pathogenic bacteria in TB patients' sputum are more diverse and complex. When a patient is infected with TB of different drug resistance types, will it affect the surrounding micro-ecological environment so that varying degrees of changes occur in species and abundance of the bacterial flora? Compared with the currently conventional diagnosis methods for phenotypic and genotyping detections, screening of TB patients with anti-TB drug resistance related microbial markers based on 16S rRNA sequencing can achieve high-throughput analysis of bacterial composition, abundance and differential flora, saving time while reducing the risk of infection during the operation. In this study, we identified biomarkers of different types of tuberculosis drug resistance from the perspective of microorganisms, so that differential distribution of respiratory flora in different types of tuberculosis drug resistance can be directly detected, thus opening a new path for the diagnosis of TB patients with anti-TB drug resistance. It is of great significance for rapid diagnostic methods with high specificity, sensitivity and low cost, and it also provides a new perspective for the study on drug resistance mechanisms of TB. Isoniazid (INH) and rifampicin (RIF) are two key first-line anti-tuberculosis drugs, so this study intends to use 16 rRNA sequencing technology to detect and analyze upper respiratory flora of TB patients with monoresistance to isoniazid (MR-INH), monoresistance to rifampin (MR-RFP), multidrug resistance (MDR), and polyresistance (PR). Potential microbial markers for different types of tuberculosis drug resistance are searched by screening differential flora, which provides certain guiding significance for drug resistance diagnosis and clinical medication of TB.

## Materials and methods

### Subjects and sample collection

The newly diagnosed 165 cases of active pulmonary TB patients in the Foshan Fourth People's Hospital and Sixth People's Hospital of Dongguan from October 2016 to March 2019 were enrolled as subjects. The criteria for enrollment of TB patients included the following: 1) *Mycobacterium tuberculosis* (Mtb) positive for sputum smear and sputum culture. 2) HIV test was negative. 3) Patients with negative p-nitrobenzoic acid (PNB) test. 4) The TB patients had never been treated with any anti-tuberculosis (anti-TB) drug.5) Mtb from TB patients had been tested for anti-TB drug susceptibility. 6) Patients agreed to participate in the study and signed informed consent. The exclusion criteria included the following: 1) patients < 16 years or > 65 years; 2) females in the gestation or lactation period; 3) patients who were alcoholic or smoked seriously; 4) patients with severe kidney diseases, heart diseases, or other diseases in a serious condition; 5) patients administered sedatives or antipsychotics; 6) patients complicated by AIDS infection, other infectious diseases, tumors, or other chronic diseases, such as diabetes mellitus; 7) patients taking immune modulators or recently receiving hormonal therapy; and 8) patients refusing to sign the informed consent form. Sputum samples from all patients were collected using a microbial protection collection tube for 16S rRNA sequencing of sputum flora. This study was approved by the ethics committees of the Dongguan Key Laboratory of Medical Bioactive Molecular Developmental and Translational Research, Foshan Fourth People's Hospital and Dongguan Sixth People's Hospital.

### Sputum flora nucleic acid extraction

Sputum sample was mixed in 1 mL protective solution(LongseeMed), then centrifuged at 12,000 rpm for 15 min to discard the supernatant. The sputum flora DNA was extracted according to the operating instructions of "Trace Bacterial Flora DNA Extraction Kit-I" (LS-R-N-007H-50/100, LongseeMed).

### Construction of sequencing library

The 16S rRNA was amplified by PCR with the kit Q5 High-Fidelity DNA Polymerase (M0491, NEB). The primer sequences of 338F were 5'-ACTCCTACGGGAGGCAGCA-3' and the 806R were 5'-GGACTACHVGGGTWTCTAAT-3'. The PCR product was detected by 2% agarose gel electrophoresis, and the target fragment was subjected to gelation recovery using the AxyPrep DNA gel recovery kit (AP-GX-50G, Axygen). Referring to the preliminary quantitative results of electrophoresis, the PCR amplified product was subjected to fluorescence quantification using Quant-iT PicoGreen dsDNA Assay Kit (P11496, Invitrogen^TM^). According to the results of fluorescence quantification, the sequencing volume of each sample requires mixing each sample in a corresponding proportion. A sequencing library was prepared using TruSeq Nano DNA LT Library Prep Kit (FC-121-4001, Illumina). BECKMAN AMPure XP Beads was used to remove self-ligated fragment of the connector by magnetic bead screening. PCR amplification was performed on the DNA fragment connected to the connector to enrich the sequencing library template. Prior to on-line sequencing, the library was subjected to quality check on an Agilent Bioanalyzer using Agilent High Sensitivity DNA Kit (5067-4626, Agilent). The library was then quantified on Promega QuantiFluor fluorescence quantification system using Quant-iT PicoGreen dsDNA Assay Kit (P11496, Invitrogen), and concentration qualified library concentration was above 2 nM. The qualified on-line sequencing libraries (index sequence was non-repeatable) were subject to gradient dilution, which were then mixed according to the required sequencing amount, and denatured into single strands by NaOH for on-line sequencing. 2×300 bp double-end sequencing was performed using a MiSeq Reagent Kit V3 (600 cycles) (MS-102-3003, Illumina) in MiSeq-PE250 sequencer.

### Sequencing data analysis

The raw data for high-throughput sequencing was first screened based on sequence quality. Quality screening was performed on double-ended sequence of FASTQ format one by one using sliding window method: the window size was 10 bp, the step size was 1 bp, and movement started from the first base position of the 5' end. The average base quality of the window is required to be ≥ Q20 (that is, the average base sequencing accuracy is ≥99%). The sequence was truncated from the first window with average mass value lower than Q20, the sequence length after truncation was required to be ≥150 bp, and ambiguous base N was not allowed. Subsequently, the double-ended sequence through mass screening was paired according to the overlapping bases using FLASH software (v1.2.7, Magoc and Salzberg, 2011). The overlapping base lengths of the two sequences of Read 1 and Read 2 were required to be ≥10 Bp, and base mismatch was not allowed. Finally, based on the Index information corresponding to each sample (i.e., Barcode sequence, a small segment of base sequence for identifying the sample at the beginning of the sequence), the connected sequence identification was assigned to the corresponding sample (complete match of Index sequence was required) to obtain effective sequence for each sample. For elimination of question sequence and sequence number statistics, question sequence was first identified using QIIME software (v1.8.0, Quantitative Insights Into Microbial Ecology, 2010). In addition to the requirement that the sequence length is ≥150 bp and ambiguous base N is not allowed, we will also eliminate: 1) sequence with 5' end primer mismatch base number > 1; 2) sequence containing consecutive identical base >8. Subsequently, USEARCH (v5.2.236) was invoked by QIIME software (v1.8.0) to examine and eliminate chimera sequences. The obtained sequences were subjected to OTU merger and division, and representative sequence of each OTU was used for classification status identification and phylogenetic analysis. Using the QIIME software, UCLUST sequence alignment tool (Edgar, 2010) was invoked for merger and OUT division of the previously obtained sequence based on 97% sequence similarity, and sequence with the highest abundance among OUT was selected as representative sequence of OTU. Then, based on the number of sequences that each OTU contains in each sample, a matrix file (i.e., OTU table) of OTU abundance in each sample was constructed. According to the abundance distribution of OTU in different samples, diversity level of each sample was evaluated, and sparse curve was used to reflect whether the sequencing depth was up to standard. For the representative sequence of each OTU, the default parameters were used in the QIIME software, and OTU representative sequence was compared with the corresponding database (Greengenes, Release 13.8) template sequence to obtain taxonomic information corresponding to each OUT. Alpha diversity analysis and Beta diversity analysis were performed using QIIME software, and the relative abundance matrix of the genus level was submitted for LEfSe analysis through the Galaxy online analysis platform (http://huttenhower.sph.harvard.edu/galaxy/). Through the PICRUSt flora metabolic function prediction tool, the existing 16S rRNA data was compared with the microbial reference genomic database with known metabolic functions to predict the metabolic function of the bacteria. The differences in 16S rRNA gene copy number of different species were simultaneously considered in the prediction process, and species abundance data in the original data was corrected for more accurate and reliable prediction results.

### Statistical analysis

All measurement data were expressed by mean ± standard deviation and analyzed by group T test, and the count data was expressed in (%) and tested by chi square test. *P* < 0.05 was considered statistically significant.

## Results

### Characteristics of the subjects

In this study, we selected 165 patients with both clinically and microbiologically confirmed active pulmonary TB. Based on drug susceptibility testing, there were 55 cases of TB patients with anti-TB drug sensitivity (DS) and 66 cases of TB patients with anti-TB drug resistance (DR). Of the DR patients, 33 cases were TB patients with MR-INH, 12 cases were TB patients with MR-RFP, 26 cases were TB patients with MDR, and 39 cases were TB patients with PR. The basic clinical information of the DR and DS patients is summarized in **Table 1**.

### Diversity analysis of sputum flora

**Figure 1:**
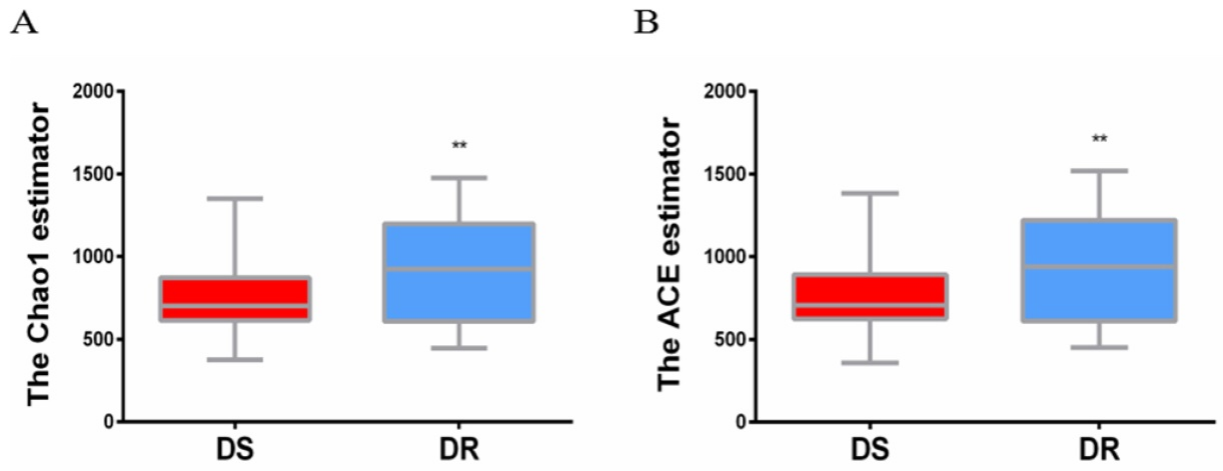
** Alpha diversity analysis of sputum flora diversity index.** (A) Chao1 index analysis of the sputum flora diversity, DR group has higher sputum flora abundance than DS group, **P < 0.01. (B) ACE index analysis of sputum flora abundance, DR group has higher sputum flora diversity than DS group, **P<0.01.

The sputum flora diversity of DS and DR patients was assessed at the OTU level. There were an average of 4507 OTUs in the DS group, 4437 OTUs in the DR group and 4147 shared OTUs. In diversity evaluation of sputum flora, Chao1 index analysis showed that Chao1 value was 759.3 ± 30.18 in DS group and 905.0 ± 41.41 in DR group. DR group had higher sputum flora abundance than DS group. ACE abundance estimation index showed that the average ACE value was 767.6 ± 31.21 in DS group and 917.0 ± 43.00 in DR group. Similarly, sputum flora abundance was higher in DR group than in DS group (**P<0.01) (**Figure 1**).

### Cluster analysis of sputum flora

**Figure 2:**
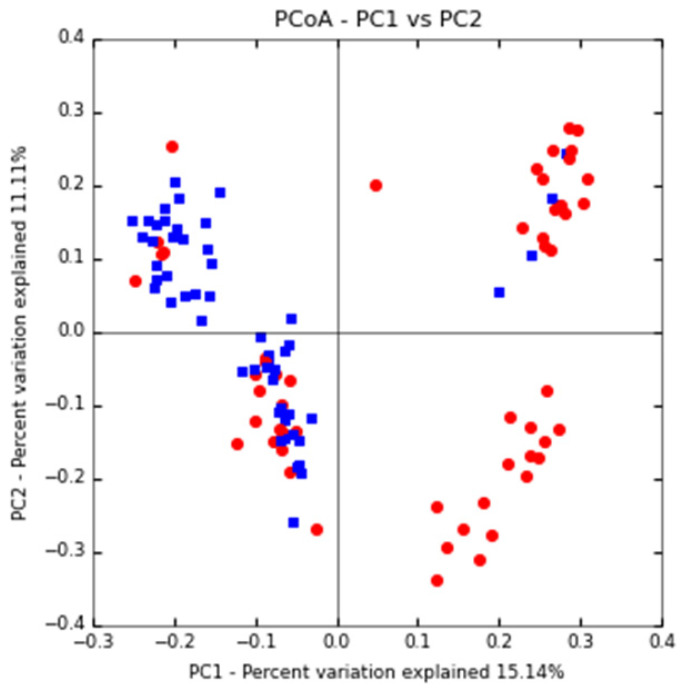
** PCoA primary coordinate analysis based on Unweighted UniFrac distance.** PC1 15.1%, PC2 11.11%. (blue: DS group, red: DR group)

Based on the PCoA principal coordinate analysis of Unweighted UniFrac distance, the phylogenetic relationship between the individual OTUs of the two groups of sputum flora was compared between DS and DR patients. **Figure 2** shows that most TB patients with DS can be distinguished from those with DR, indicating certain difference in the flora structure between the two groups.

### Analysis of sputum flora composition

**Figure 3:**
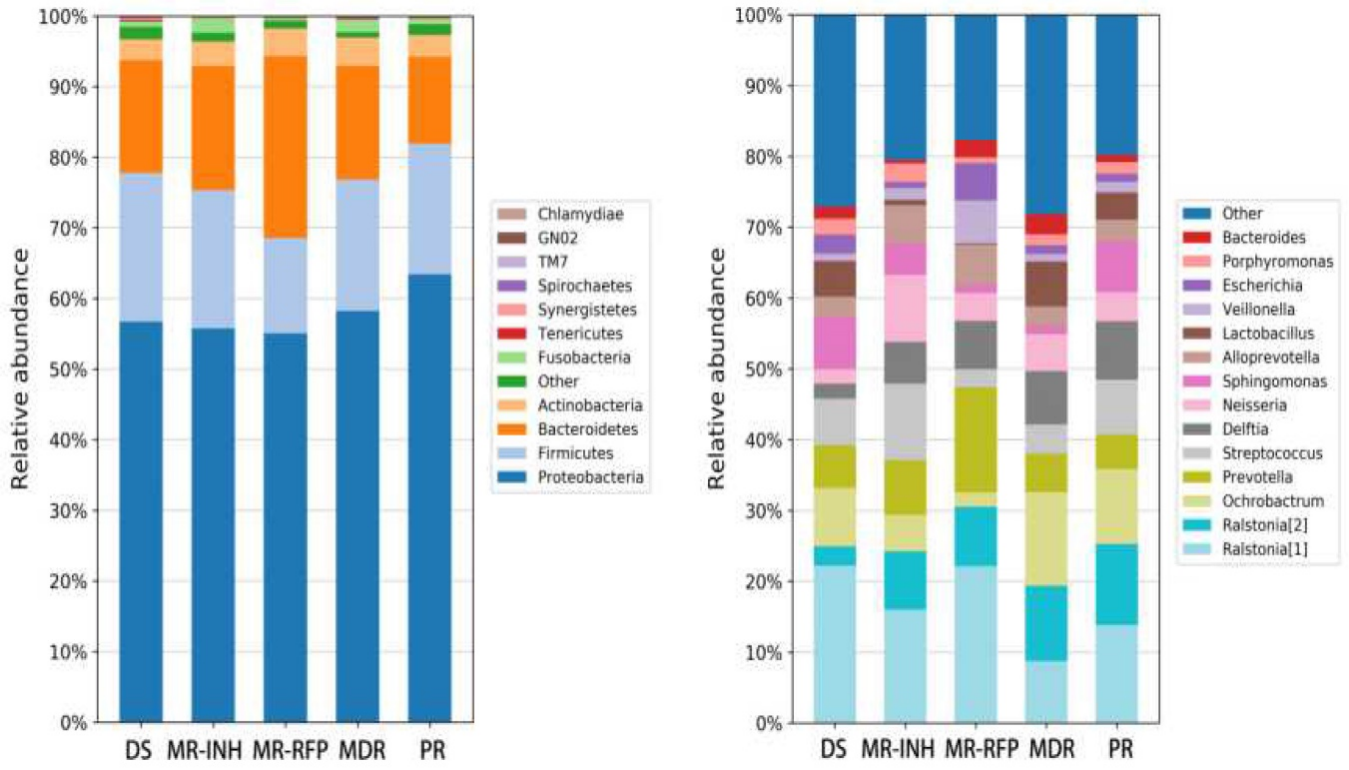
**Comparison of the relative abundance of major bacteria in each group of samples.** (A) The flora in human sputum is mainly composed of Proteobacteria, Firmicutes, Bacteroidetes, and Actinobacteria. (B) The human sputum sample mainly contains 14 bacterial genera, Ralstonia of the Ralstoniaceae family, Ralstonia, Ochrobactrum, Prevotella, Streptococcus and Delftia of the Burkholderiaceae family account for about 50% of all the bacterial genera.

Proteobacteria, Firmicutes, Bacteroidetes, Actinobacteria and Fusobacteria are the most important bacteria, accounting for more than 97% of the sputum flora. Where, Proteobacteria accounts for more than 50% of the flora, and other bacteria such as Teneriquetes, Synergistete, GNO2, Spirochaetes, TM7 and Chlamydiae were detected with a relative abundance of <1%. The proportion of proteobacteria did not change much in the DS, MR-INH, and MR-RFP groups but increase in the MDR and PR groups. The proportion of proteobacteria was higher in patients with MDR than in patients with drug sensitivity and single drug resistance (**Figure 3A**).

At the genus level, the sputum samples covered 14 major bacterial genera. Ralstonia (8.28%), Ochrobactrum (7.85%), Prevotella (7.77%), and Streptococcus (6.36%), Delftia (6.12%) in the Burkholderiaceae family accounted for about 50% of all bacterial genera. Compared with the DS group, the ratio of Ralstonia, Delftia, Neisseria in Burkholderiaceae family was up-regulated in all DR (MR-INH, MR-RFP, MDR, PR) groups. Comparison between single-drug resistant (MR-INH, MR-RFP) group and two-drug-resistant (MDR, PR) group, the ratio of Ralstonia, Prevotella, Alloprevotella, and Veillonella in the Ralstoniaceae family was down-regulated, and the ratio of Ralstonia, Ochrobactrum, and Lactobacillus in the Burkholderiaceae family was up-regulated (**Figure 3B**). It is worth mentioning that Mycobacterium accounts for a very small proportion of the entire sputum microbiota, whose relative abundance is only 0.06%.

### Differential flora analysis

**Figure 4:**
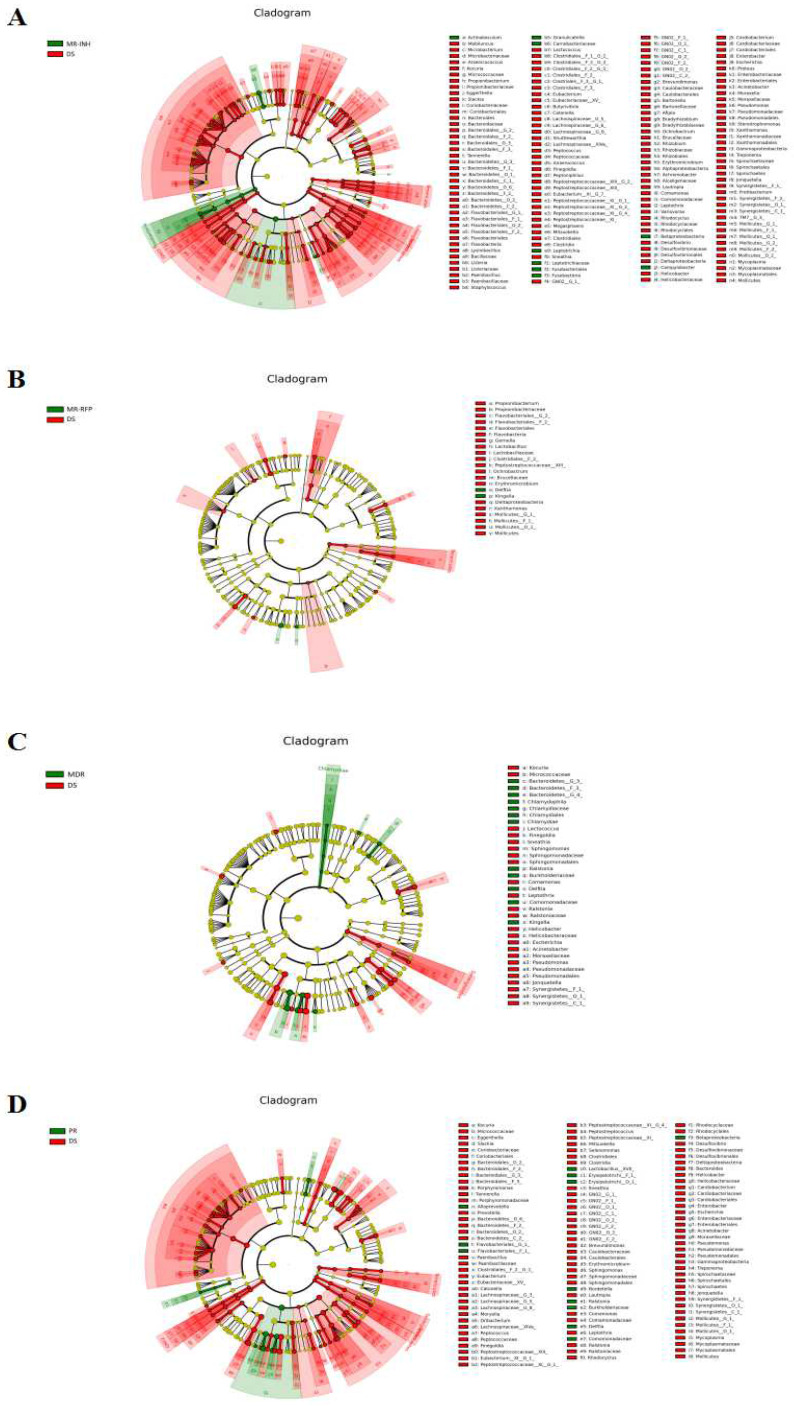
** LEfSe analysis of sputum flora differences between samples. (A)** Differential display of flora in MR-INH and DS groups. **(B)** Differential display of flora in MR-RFP and DS groups. **(C)** Differential display of flora in MDR and DS groups.** (D)** Differential display of flora in PR and DS groups. The classification level tree displayed by cladogram describes the hierarchical relationship of all the flora from phylum to genus (successively ordered from the inner circle to the outer circle) in the sample community. The node size corresponds to the average relative abundance of the flora, red and green respectively indicate flora with high abundance, and the difference is significant. The letters make the names of the flora with significant differences between the groups.

**Figure 5:**
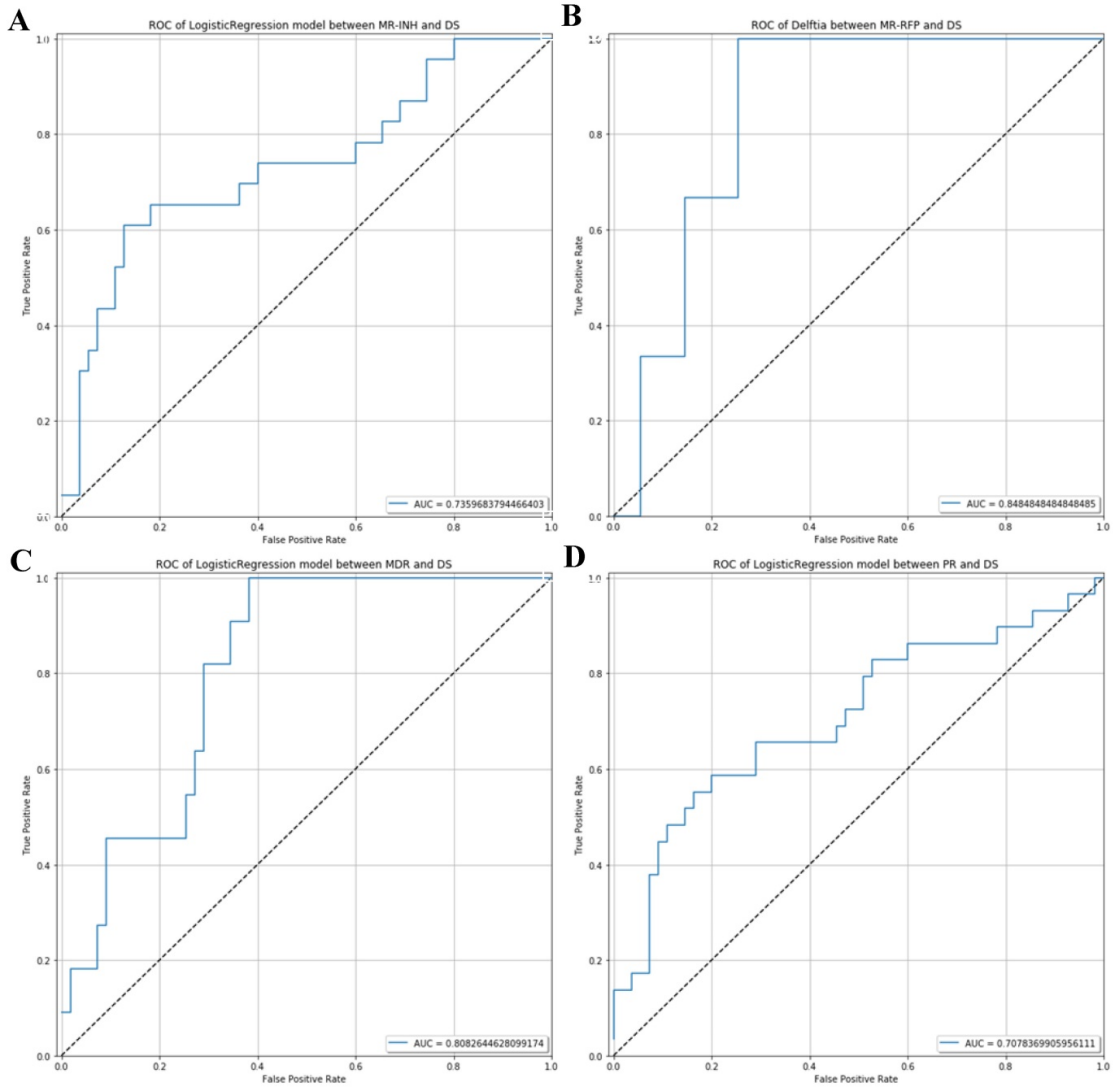
** ROC analysis which can be used to distinguish potential diagnostic flora of respiratory microecology in the drug-sensitive group and different drug-resistant groups.** (A) ROC curve between MR-INH and DS. (B) ROC curve between MR-RFP and DS.(C) ROC curve between MDR and DS. (D) ROC curve between PR and DS.

Based on LEfSe analysis, the differential flora of MR-INH, MR-RFP, MDR, PR and DS groups were screened separately. At the genus level, the 3 bacterial genera of Leptotrichia, Granulicatella, and Campylobacter were significantly higher in the MR-INH group than in the DS group (P<0.05, **Figure 4A, Table 2**). One bacterial genus of Delftia was significantly higher in MR-RFP group than DS group (P<0.05, **Figure 4B, Table 3**). The 6 bacterial genera of Delftia, Kingella, Ralstonia, Chlamydophila were significantly higher in MDR group than in DS group (P < 0.05, Figure 4 C, Table 4). Delftia, Ralstonia and Bordetella were significantly higher in PR group than in DS group (P < 0.05, **Figure 5D, Table 5**). Flora function prediction revealed that flora composition of DR group was closely related to the digestive system, circulatory system and immune system diseases, with the related flora abundance higher than that in the DS group. We speculate that the three bacterial genera of Leptotrichia, Granulicatella, and Campylobacter are potential diagnostic markers for single-resistance INH, Delftia is a potential diagnostic marker for single-resistance RFP, Kingella and Chlamydophila are potential diagnostic markers for multi-drug resistance, and Bordetella is a potential diagnostic marker for multidrug-resistance.

The AUC value of ROC curve MR-INH, MR-RFP, MDR and PR was 0.7359, 0.8485, 0.8083 and 0.7078 respectively. In consequence, by random forest prediction model, the results indicated that differential microorganisms could be the potential marker to differentiate the MR-INH, MR-RFP, MDR, PR patients as shown in **Figure 5**.

## Discussion

Compared with the traditional study on single pathogens, investigating the factors affecting disease from different perspectives can provide a deeper understanding of the pathogenic mechanism. Many studies have shown that certain diseases are associated with imbalances in microbial communities rather than the presence of a single pathogenic pathogen^2^-^6^. At present, there are relatively few studies on TB microbiology in China, most of which are preliminary investigations on respiratory flora of TB patients and normal population, while respiratory micro-ecology of TB infections of different drug-resistant types is rarely reported.

Wu et al. found that Streptococcus, Gramulicatella and Pseudomonas were higher abundance in TB patients, while Prevotella, Leptotrichia, Treponema, Catonella and Coprococcus were higher in healthy groups after 16S RNA sequencing analysis of sputum flora in 25 cases new-onset TB patients, 30 cases recurrent TB patients, 20 cases failed TB patients and 20 healthy controls in Shanghai^6^. Furthermore, they also found that the frequency and abundance of Bulleidia and Atopobium were lower in patients with recurrent pulmonary TB than in new-onset TB patients, while the proportion of Pseudomonas/Mycobacterium was higher in recurrent TB patients than in new-onset TB patients, and the proportion of Treponema/Mycobacterium was higher in new-onset TB patients, indicating that destruction of these bacteria may be a risk factor for TB recurrence^6^. Notably, Wu et al. found that Pseudomonas was more frequently present in patients with failed treatment than in cured new-onset TB patients, and the proportion of Pseudomonas/Mycobacterium is higher in patients with failed treatment than in new-onset TB patients^6^. Therefore, lung microbial diversity test means great significance for the treatment and recurrence risk assessment of TB patients.

Herein, we sequenced the 16S rRNA V4-V5 region of sputum flora in TB patients by MiSeq-PE250, and reported the difference in microbial composition of lower respiratory tract in patients with MR-INH, MR-RFP, MDR, PR and DS TB for the first time. Saliva and pharyngeal secretions could reflect the flora composition of the lower respiratory tract^8^, so we chose sputum samples in this study. The best sample is lung lavage fluid, which can reflect the flora composition of the lower respiratory tract. However, sampling of lung lavage fluid is difficult because it makes the subject feel pain and causes lung damage. On the contrary, sputum samples are readily available by non-invasive sampling method. In our study, we detected that human sputum samples were mainly covered by four major bacteria phylum: Proteobacteria, Firmicutes, Bacteroidetes and Actinobacteria. It is reported that these four bacteria are related to the diversity of the sputum flora of TB patients^3^. In addition, these four types of bacteria are also the main flora present in the human mouth, skin and colon^9^. Alpha diversity analysis showed that composition diversity of lower respiratory tract flora was higher in TB patients with DR than in TB patients with DS. Research on flora diversity showed that sputum flora diversity was higher in TB patients than in healthy individuals^4^. The lower respiratory tract represents an open system that freely communicates with the environment. We speculated that in TB patients, lung microenvironment may become more susceptible to colonization of some foreign microorganisms. Under normal health conditions, the host must constantly distinguish symbionts and pathogens by establishing appropriate adaptive immune responses, producing local inflammatory damage during powerful clearance of mycobacterium TB, which may increase the colonization of foreign pathogenic bacteria in the lungs, especially in immunosuppressed subjects^10^. It was found in previous studies that TB patients with DR had a lower innate immune function than TB patients with DS^11^,^12^ , which may be the reason why the sputum flora diversity is higher in TB patients with DR than in TB patients with DS. Tissue damage caused by these responses will result in extensive fibrosis and recurrent infection, which may reduce the pathogen clearance of lymph and lymph-associated particles in the infected area^13^. Deletion of the respiratory microbiota, including the invasion of foreign pathogens, or the reduction of probiotics may result in the destruction of immune balance, leading to disease activation, vulnerability to new infections^14^,^15^ or interference with cure of the disease. We found that flora diversity was higher in multi-drug resistance and multidrug resistance groups than in single-drug resistance group when compared flora diversity of TB patients with DS, MR-INH, MR-RFP, MDR, and PR.

LEfSe analysis found that there were 3 genera with significantly increased abundance in the TB patients with MR-INH, 1 genus with significantly increased abundance in the TB patients with MR-RFP, 4 genera with significantly increased abundance in the TB patients with MDR, and 3 genera with significantly increased abundance in the TB patients with PR. By observing the unique genus in each group, we screened diagnostic markers for multiple drug-resistant types of tuberculosis from genera of Leptotrichia, Granulicatella, Campylobacter, Delfitia, Kingella, Chlamydophila, Bordetella. The flora function prediction revealed that these genera are closely related to digestive system, circulatory system and immune system diseases.

Leptotrichia is normally colonized in the oral cavity^16^ and reproductive tract^17^, which belongs to the normal flora of humans as anaerobic bacteria. However, in rare cases, Leptotrichia is isolated and cultured from the blood of patients with oral mucosal lesions such as periodontitis^18^, which usually spread into the blood when the mucosal barrier is destroyed. Leptotrichia is also found in peritoneal fluids and blood of patients with immunodeficiency such as neutropenia, HIV, leukemia and endocarditis and it is considered as potential pathogens in patients with neutropenia^19^. It is reported that Leptotrichiaceae is much higher in bovine lung and lymph node tissues with respiratory diseases than in healthy cattle without lung damage, so it has been suggested that this family may be associated with human lung disease. A clinical study identified Leptotrichia from lung lavage of an elderly pneumonia patient, which was presumed as the cause of the disease^20^. The role of Leptotrichia in disease has rarely been reported mainly due to the difficulty of its isolation and identification, and it is considered to be an emerging pathogen^21^,^22^ Granulicatella, a facultative anaerobic Gram-positive cocci, was originally known as nutrientally variant streptococci (NVS), which constitutes the normal flora of human oral, upper respiratory, genitourinary and gastrointestinal tracts^23^. However, it can lead to infections in other parts of the body, and Granulicatella adiacens were associated with infective endocarditis, bacteremia, ophthalmic infections, prosthetic joint infections, septic arthritis and central nervous system infections^24^,^25^. Campylobacter, an anaerobic bacteria genus found in human mouth, is commonly found in plaque under the gums of patients with periodontitis^26^. However, campylobacter curvus from pleural effusion of a patient with empyema by 16S rRNA sequencing and PCR and found that Campylobacter genus in the mouth is relevant with abscess in the respiratory system. Moreover, empyema usually occurs in patients with poor oral hygiene^27^. Campylobacter rectus causes empyema, who considered that Campylobacter is the pathogen of respiratory abscess^28^. Delftia is a non-fermenting Gram-negative bacterium found in deep bronchial aspirated liquid and blood cultures of patients with chronic obstructive pulmonary disease (COPD), which can also cause sepsis. Clinically, pleural blood pus was taken via percutaneous puncture from a patient with chronic empyema complicated with low immune function, and Delftia acidovorans was found by 16S rRNA sequencing^29^. Kingella is a Gram-negative coccus which is increasingly regarded as an invasive pediatric pathogen. Romain's study found that the infection of Kingella kingae osteoarticular (KKO) is usually associated with infection of the upper respiratory tract. Children infected with KKO had higher respiratory virus in the oropharynx than children without KKO^30^. Chlamydophila is a cytozoic Gram-negative bacterium which can cause various infections such as pneumonia, sinusitis, bronchitis, rhinitis, chronic obstructive pulmonary disease (COPD) or asymptomatic infection^31^. Chlamydophila's lung infection is associated with increased asthma and lung cancer. In particular, when smokers are infected with Chlamydophila, plasmacytoid DCs (pDCs) in the lungs will inhibit response preference of Chlamydophila-induced bone marrow dendritic cell mDCs (myeloids, DCs) to Th2, so that lung immune environment is in suppressed state^32^. Nine species of Bordetella have been identified to date and all of them are associated with respiratory infections in humans and other mammals. Whooping cough is a highly contagious human respiratory disease caused by B. pertussis Gram-negative pathogens^33^. B. bronchiseptica occurs in immunocompromised or immunodeficient patients with pneumonia, endocarditis, peritonitis, meningitis, sepsis, recurrent bacteremia.

Herein, we found that pulmonary micro-environment of TB patients is more susceptible to colonization of external pathogens and infection of different drug-resistant Mtb leads to different flora changes. The occurrence of TB symptoms may be a result of interaction of different floras. For the most of the differential genus, we know very little about their function except the extreme difficulty in its isolation and identification. Based on 16S rRNA sequencing technology, we can detect its presence at the molecular level and it needs further investigation on the mechanism between the floras and drug resistance of TB.

In conclusion, seven novel microorganisms (Leptotrichia, Granulicatella, Campylobacter, Delfitia, Kingella, Chlamydophila, Bordetella) were identified by 16S rRNA sequencing as diagnostic markers for different drug resistance types of TB. Leptotrichia, Granulicatella, Campylobacter are potential diagnostic marker for indication of TB patients with single-resistance INH. Delftia is a potential diagnostic marker for indication of TB patients with single drug-resistance RFP. Kingella and Chlamydophila can be used as diagnostic markers for indication of TB patients with PR. Bordetella can be used as a potential diagnostic marker for identification of TB patients with MDR.

## Figures and Tables

**Table 1:** Basic information for clinical samples included in this trial

	DS-TB	MR-INH	MR-RFP	MDR	PR
Number (%)	55 (45.45%)	33 (20.00%)	12 (7.27%)	26 (15.76%)	39 (23.64%)
Age (median±SD, range)	37.45±13.89 (16-67)	43.61±14.16 (19-62)	42.67±6.429 (38-50)	40.73±14.77 (21-68)	43.96±12.08 (23-63)
Male	37 (67.27%)	22 (66.67%)	7 (58.33%)	16 (61.54%)	25 (64.10%)
Female	18 (32.73%)	11 (33.33%)	5 (41.67%)	10 (38.46%)	14 (35.90%)

**Table 2:** Relative abundance of differential genus in MR-INH and DS groups

Identity	Relative abundance (x±s, %)		p value
	DS	MR-INH	
Leptotrichia	0.51±0.01	1.70±2.32	0.0016
Granulicatella	0.81±0.01	2.32±0.04	0.0013
Campylobacter	0.02±0.00	0.06±0.00	0.0014

**Table 3:** Relative abundance of differential genus in MR-RFP and DS groups

Identity	Relative abundance (x±s, %)		p value
	DS	MR-RFP	
Delftia	2.12±0.05	6.82±0.10	0.03

**Table 4:** Relative abundance of differential genus in MDR and DS groups

Identity	Relative abundance (x±s, %)		p value
	DS	MDR	
Delftia	2.12±0.05	7.54±0.07	0.0023
Kingella	0.02±0.00	0.06±0.00	0.0046
Ralstonia	2.71±0.10	10.63±0.10	0.0187
Chlamydophila	8.249e-004±0.00	3.708e-003±0.00	0.0329

**Table 5:** Relative abundance of differential genus in PR and DS groups

Identity	Relative abundance (x±s, %)		p value
	DS	PR	
Delftia	2.12±0.05	8.25±0.08	< 0.0001
Ralstonia	2.71±0.10	11.44±0.12	0.0005
Bordetella	1.155e-003±0.00	0.02±0.00	0.0324
